# Enhancing RCPsych Addiction Competencies: A Pilot Programme Integrating Training and Taster Days for North London Foundation Trust’s Core Trainees

**DOI:** 10.1192/bjo.2025.10320

**Published:** 2025-06-20

**Authors:** Munzir Quraishy, Harrison Howarth, Chern Wong, Yathooshan Ramesh, Kevin O’Neill

**Affiliations:** North London Foundation Trust, London, United Kingdom

## Abstract

**Aims:** There are new requirements for addictions competencies as part of the 2022 core trainee curriculum. The Silver Guide, section 5.1.2 includes a requirement for two work based placed assessments (WBPA) in addictions to be completed during core training. This has posed challenges nationally, especially in England due to the way addiction services are commissioned and tendered. Whilst most training schemes have opted for CBD groups, our region of London wanted to provide practical and experiential learning with taster days at both NHS and 3rd sector services. Trainees were an integral part of the stakeholder group to ensure co-production.

**Methods:** Between Nov 23 and March 24, a pilot training lecture day and 4 taster days were held. Trainees registered on a first-come-first-served basis. The taster days gave trainees an opportunity to assess at least one patient under supervision in an addictions environment and have a case-based discussion (CBD) with an addictions consultant. The teaching day included presentations across addiction topics requested by the trainees. Feedback, both quantitative and qualitative was collated after both events.

**Results:** 100% of trainees felt more confident in assessing patients with substance use problems and 88% felt more confident in management after attendance. 100% of trainees also felt that the programme gave them useful knowledge in their future practice beyond what could be offered by other sources. 100% of trainees also felt the topics were relevant and would recommend the course to others. Trainees described the taster days as providing “intensive, immersive and enriching experience”, and that 1 trainee each day “was the right level”. It was also described as “a really great learning opportunity” that they “would do again”.

**Conclusion:** Both the training day and taster days had overwhelmingly positive feedback. This has led to a permanent implementation of a rotating programme between the 3rd sector and NHS addiction services in the region. Particularly with the new curriculum requirements, these appear to fill a gap in trainee learning. This programme is the first of its kind in the country, as other trusts have opted to run CBD groups. The aim will be to expand this into an annual programme for all core trainees in the trust, continually improve on the structure and possibly expand attendance to higher grades. This year, many doctors of varying grades, including other consultants have expressed interest in attending. Widening addiction training will only improve patient care nationally.

